# The T‐Box Transcription Factors TBX2 and TBX3 Are Molecular Targets of Piroctone Olamine in the Treatment of Pancreatic Cancer

**DOI:** 10.1111/jcmm.70736

**Published:** 2025-07-27

**Authors:** Karabo Serala, Sanele Mdletshe, Jinming Bai, Amaal Abrahams, Odile Gayet, Loic Moubri, Nelson Dusetti, Sharon Prince

**Affiliations:** ^1^ Department of Human Biology, Faculty of Health Sciences University of Cape Town, Observatory Cape Town South Africa; ^2^ Cancer Research Center of Marseille, Inserm, Paoli‐Calmettes Institut Aix‐Marseille University, Scientificand Technological Park of Luminy Marseille France

**Keywords:** drug repurposing, piroctone olamine, targeted therapy, TBX2, TBX3

## Abstract

Pancreatic ductal adenocarcinoma (PDAC) has a poor 5‐year survival rate of < 10% and its incidences are continuously rising worldwide. This highlights an urgent need for effective therapies to reduce its burden. Repurposing commercially available non‐cancer drugs that inhibit key drivers of PDAC may facilitate the rapid identification of effective drugs. In PDAC, the expression of the TBX2 and TBX3 transcription factors correlates with metastasis and poor patient survival. This study showed that when TBX3 was depleted in 2D and 3D PDAC cell culture models, the cells underwent senescence and had reduced proliferative ability and spheroid growth. Interestingly, TBX2 levels increased in shTBX3 cells and depleting TBX2 in these cells inhibited their migration. Our results thus demonstrated that TBX2 and TBX3 have distinct oncogenic functions and that any effective anti‐PDAC drug must inhibit them both. The antifungal piroctone olamine, previously identified as a TBX2‐/3‐targeting drug in melanoma and rhabdomyosarcoma, inhibited the levels of TBX2 and TBX3 and recapitulated the phenotypes observed when they were knocked down in 2D and 3D PDAC cell culture models. Impressively, piroctone olamine was also effective in PDAC patient‐derived organoids. Together, our data demonstrate the potential of piroctone olamine to be repurposed for treating TBX2‐/3‐dependent PDAC.

## 1 Introduction

Worldwide in 2020, pancreatic cancer accounted for 495,773 new cancer cases and 466,003 cancer deaths [1]. Pancreatic ductal adenocarcinoma (PDAC) constitutes 90% of all pancreatic cancer cases, has a 5‐year survival rate of < 10% and ranks seventh in cancer‐related mortalities [2]. Furthermore, PDAC is predicted to become the second leading cause of cancer‐related deaths worldwide by 2030 [2, 3]. Surgical resection is the only means of cure for PDAC, but only 20% of patients are eligible [4]. The remaining 80% of PDAC patients present with locally advanced or metastatic disease, and they are treated with gemcitabine (GEM), FOLFIRINOX or GEM plus nab‐Paclitaxel [5]. However, these regimens exhibit indiscriminate cytotoxicity and are associated with high rates of tumour recurrence and relapse [2, 5]. Targeted therapies hold great promise as an alternative cancer treatment strategy; however, the efforts to target genetic drivers of PDAC have resulted in drugs that benefit only a small proportion of patients [3, 6]. It would, therefore, be advantageous to identify molecular targets present in most PDAC patients.

The T‐box factor‐3 (TBX3) is a member of the T‐box gene family that encodes a group of ancient and evolutionarily conserved transcription factors primarily involved in embryonic development [7]. Importantly, while mutations resulting in reduced levels of TBX3 give rise to the ulnar mammary syndrome, high levels of TBX3 expressed postnatally impact several oncogenic processes including bypassing senescence and apoptosis, promoting substrate‐dependent and independent cell proliferation, cell migration and invasion and in vivo tumour‐forming ability [7]. TBX3 is expressed in the developing pancreas and the exocrine cells of the mature pancreas, and its levels increase in PDAC tissues, which directly correlate with distant metastasis and poor patient survival [8, 9]. Perkhofer et al. showed that PDAC cells overexpressing TBX3 had an increased ability to form tumours and metastasise [10]. While these findings suggest that targeting TBX3 may be a promising therapeutic approach for treating PDAC patients, the impact of biologically inhibiting TBX3 on the PDAC phenotype has not been explored.

Piroctone olamine (PO) is an antifungal agent used in cosmetic and oral care products as well as in hair products such as antidandruff shampoos [11]. To achieve its antifungal activity, PO forms complexes with iron and subsequently inhibits energy metabolism and oxygen absorption in the mitochondria of fungi [12, 13]. PO has also been explored for the treatment of cancer and was shown to induce apoptosis in myeloma and lymphoma cells, and when loaded on Fe_3_O_4_ nanoparticles, it inhibited the activity of matrix metalloproteinase‐2 in human fibrosarcoma cells [11, 14]. Furthermore, it was shown to inhibit proliferation and induce apoptosis of glioma cells by interfering with mitochondrial dynamics by inhibiting the PI3K/AKT pathway [15]. PO was also shown to inhibit cell viability in several cancer cell lines, including two PDAC cell lines and was recently reported to inhibit TBX3, and its homologue TBX2, and to exert anti‐cancer activity in TBX2‐/3‐dependent melanoma and rhabdomyosarcoma [16]. However, there is still a paucity of information regarding its anti‐cancer activities and mechanisms of action in PDAC and whether it inhibits TBX2/3 proteins in PDAC is not known [17, 18, 19].

Here, we showed that TBX2 and TBX3 play distinct and important oncogenic roles in PDAC and that targeting them simultaneously may hold important therapeutic benefits for PDAC. We further showed that PO inhibits TBX2 and TBX3 levels and exhibits promising anti‐PDAC activity in 2D and 3D cell cultures and patient‐derived organoids. Overall, this study provided compelling evidence that PO may be a promising TBX2‐/3‐targeting drug that can be repurposed for the treatment of TBX2‐/3‐dependent PDAC.

## 2 Materials and Methods

### 2.1 Analysis of the Cancer Genome Atlas PDAC Cohort (TCGA‐PAAD) and Cancer Cell Line Encyclopaedia (CCLE)

The expression and clinical significance of *TBX3* in PDAC patient samples were investigated by analysing the TCGA‐PAAD gene expression data via the Gene Expression Profiling Interactive Analysis (GEPIA) platform (https://gepia.cancer‐pku.cn/). PDAC cell lines that express *TBX3* were identified by analysis of the CCLE (https://sites.broadinstitute.org/ccle/).

### 2.2 Cell Culture

PANC‐1, BxPC‐3, SW1990 and FG0 cells were cultured in Dulbecco's Modified Eagle Medium and CFPAC‐1 cells in Iscove's Modified Dulbecco's Medium under conditions described previously [20].

### 2.3 Cell Treatments

PO and GEM (Sigma Aldrich, USA) were dissolved in dimethyl sulfoxide (DMSO) to achieve a stock concentration of 5 mM and stored at –20°C. The drugs were diluted to the desired concentration(s) using the growth media, and the percentage of DMSO in the highest drug concentration was used as a vehicle control.

### 2.4 Plasmids, Small‐Interfering RNA and Cell Transfection

Cells were transfected with DNA constructs (pPB‐hCMV1‐cHApA‐Empty‐3xFLAG, pPB‐hCMV1‐cHApA‐Tbx3‐3xFLAG and TBX2 luciferase reporter) or siRNAs using the XtremeGENE HP (Roche, Switzerland) or HiPerfect (QIAGEN, Germany) transfection reagents, respectively, following the manufacturer's instructions.

### 2.5 Generation of Stable Cell Lines

PANC‐1 cells were stably transfected with the pSuper.neo/GFP expression vector containing a sequence targeted to TBX3 (shTBX3) or a non‐specific control (shCtrl) as previously described [21].

### 2.6 Western Blot Analysis

Total protein lysates were prepared and subjected to western blot analysis as previously described [22]. The antibodies and dilutions used are shown in Table S1.

### 2.7 Quantitative Real‐Time PCR (qRT‐PCR)

qRT‐PCR was performed as previously described [23]. The Light Cycler 2.0 system (Roche, Switzerland) and primers to human *TBX3* (QT00022484), *TBX2* (QT00091266) and *GUSB* (QT00046046) from Qiagen (Maryland, USA) were used. The results were analysed using the 2^−∆∆Ct^ method and data were normalised to *GUSB* levels.

### 2.8 Immunofluorescence

Immunofluorescence was performed as previously described [24]. The antibodies and concentrations used are shown in Table S2.

### 2.9 Cell Proliferation Assay

Cell proliferation was measured by growth curve analysis at 2‐day intervals for 6 days.

### 2.10 Cellular Senescence Assay

The Senescence β‐Galactosidase Staining Kit (#9860; Cell Signalling Technology, USA) was used according to the manufacturer's instructions.

### 2.11 Cell Migration Assay

Cell migration was assessed using the in vitro 2D scratch motility assay as previously described and to inhibit cell proliferation, 10 μM Mitomycin C (Sigma Aldrich, USA) was added at the same time as PO or GEM [25].

### 2.12 Cell Invasion Assay

Cell invasion was investigated using the Transwell cell invasion assay as previously described [20].

### 2.13 3D Spheroid Growth, Viability and Invasion Assays

PDAC spheroids were generated and their growth, viability and invasion were assessed as previously described [20].

### 2.14 Luciferase Reporter Assays

Cells were transfected for 48 h with 200 ng of the TBX2‐luciferase construct and 100–300 ng of Flag‐TBX3 expression vector or empty vector using the XtremeGENE HP transfection reagent (Roche, Switzerland). The pRL‐TK vector containing a thymidine kinase promoter driving expression of a renilla reporter was included as an internal control for transfection efficiency (20 ng per condition). The firefly and renilla luciferase activities were determined using the Dual‐Luciferase Reporter Assay System (Promega, USA) following the manufacturer's instructions and measured using a Luminoskan Ascent luminometer (Thermo Labsystems, USA).

### 2.15 Cell Viability Assays

The viability of PDAC and FG0 skin fibroblasts post treatment with PO or GEM was measured using the MTT assay as previously described [25]. Selectivity indices (SI) were calculated as a ratio of the half maximal inhibitory concentrations (IC_50_) of FG0 and PDAC cells.

### 2.16 Patient‐Derived Organoids Viability Assay

Patient‐derived organoids (PDOs) were established and cultured as previously described [26]. Their morphology was assessed by light microscopy (EVOS M5000 Imaging System; ThermoFisher Scientific, USA) and their viability was measured using the CellTiter‐Glo 3D Cell Viability Assay kit (Promega Corporation, USA) according to the manufacturer's instructions.

### 2.17 Colony Formation Assays

Clonogenic assays were performed as previously described [25].

### 2.18 Cell Cycle Analysis

Cell cycle analysis was performed as previously described [25].

### 2.19 Apoptosis Assays

Apoptosis was measured using the FITC Annexin V/Dead Cell Apoptosis kit (V13242; Thermo Fisher Scientific, Massachusetts, USA) according to the manufacturer's instructions.

### 2.20 Statistical Analysis

Unless otherwise stated, data were obtained from at least three independent experiments and analysed by a parametric unpaired *t*‐test. Error bars represent the standard error of the mean (SEM), and significance was accepted at *p < 0.05, **p < 0.01, ***p < 0.001 and ****p < 0.0001.

## 3 Results

### 3.1 TBX3 Is Expressed at High Levels in PDAC Tissues and Cell Lines

**FIGURE 1:**
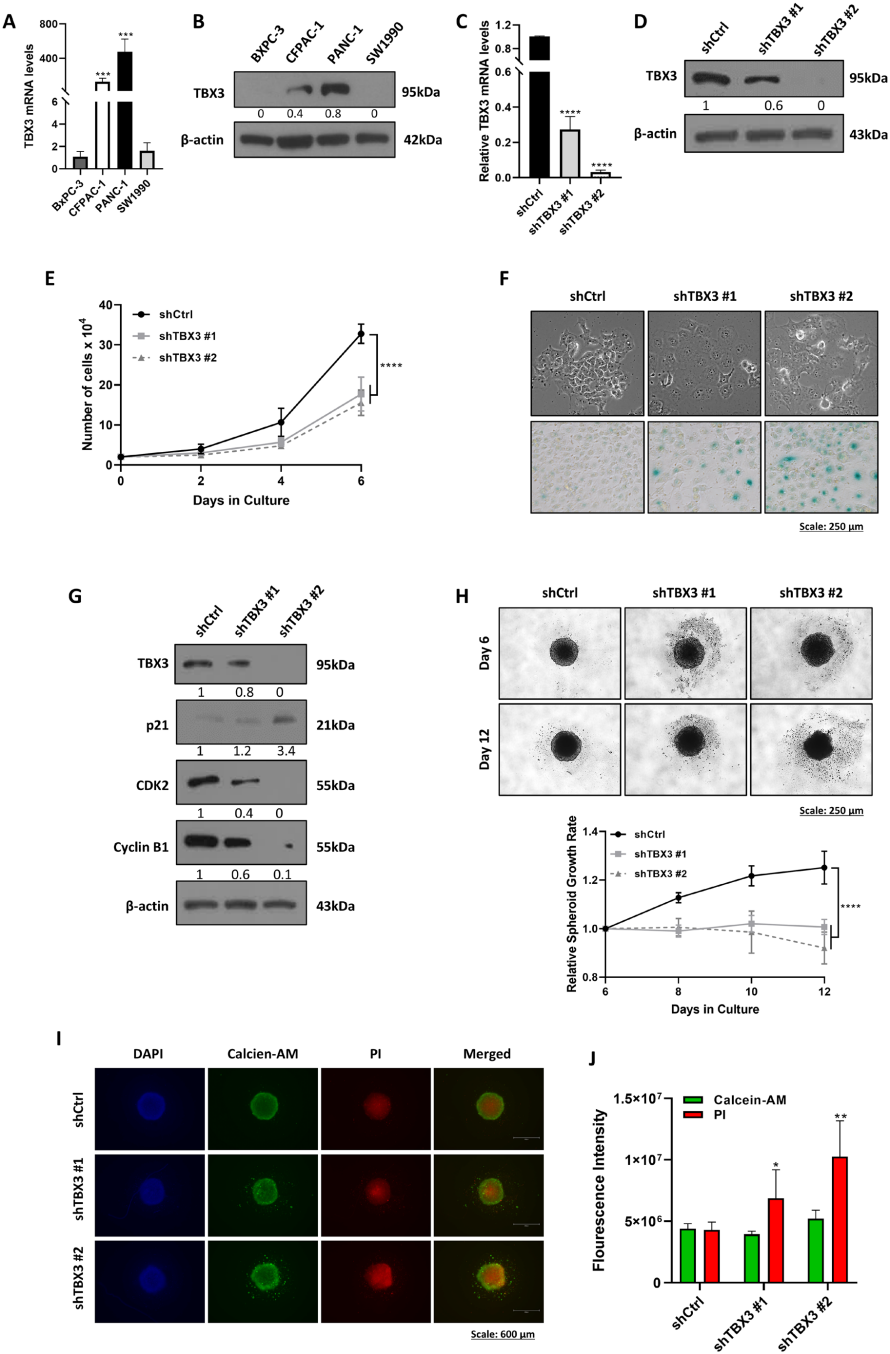
Depleting TBX3 inhibits the proliferation of PDAC cell cultures. (A) qRT‐PCR analysis of *TBX3* levels in BxPC‐3, CFPAC‐1, PANC‐1 and SW1990 PDAC cell lines. GUSB was used as a housekeeping gene, and data were expressed relative to BxPc‐3 cells. (B) Western blot analysis of TBX3 levels in the cell lines described in (A). (C) qRT‐PCR analysis of *TBX3* levels in the shCtrl, shTBX3 #1 and shTBX3 #2 cells that were used as models in the study. GUSB was used as a housekeeping gene, and data were expressed relative to shCtrl cells. (D) Western blot analysis of TBX3 levels in cells described in (C). (E) Growth curve analysis of the shCtrl, shTBX3 #1 and shTBX3 #2 cells performed over 6 days. (F) Upper Panel: light microscopy images (10× magnification; scale bar: 250 μm) of shCtrl, shTBX3 #1 and shTBX3 #2 cell morphology. Lower Panel: Representative brightfield microscopy images of shCtrl, shTBX3 #1 and shTBX3 #2 cells after senescence‐associated β‐galactosidase staining. (G) Western blot analyses of the cell cycle regulators p21, CDK2 and Cyclin B1 in shCtrl, shTBX3 #1 and shTBX3 #2 cells. (H) Representative light microscopy images (10× magnification; scale bars: 250 μm) of shCtrl, shTBX3 #1 and shTBX3 #2 spheroids. The accompanying graph shows the growth rate of the spheroids over 12 days which was determined by dividing the size of the spheroids on day 12 by the spheroid sizes measured on day 6 and the size of the spheroids at day 6 is set to 1 on the graph. (I) Representative fluorescence microscopy images (4× magnification; scale bars: 600 μm) of the spheroids in (H) stained with Calcein‐AM (live cells, green), propidium iodide (dead cells, red) and DAPI on day 12. (J) Quantitation of Calcein‐AM and propidium iodide fluorescence intensity from the images in (I). For all western blots, β‐actin was used as a loading control and densitometric readings were expressed relative to shCtrl cells. Data were analysed using the parametric unpaired *t*‐test by the GraphPad Prism 8.0 software. *p < 0.5, **p < 0.01, ***p < 0.001, ****p < 0.0001; error bars represent mean ± SEM of three independent biological repeats.

To obtain a global overview of the expression and clinical significance of TBX3 in a large cohort of PDAC patients, we analysed the RNA‐sequencing data from TCGA‐PAAD. The results revealed that *TBX3* levels were significantly higher in PDAC tissues compared to normal tissue (Figure S1A) and that there was an inverse correlation between *TBX3* levels and overall patient survival (Figure S1B). To identify PDAC cell lines that express high levels of TBX3, the CCLE was analysed, and the data revealed that TBX3 is expressed in 27 of 46 PDAC cell lines including the PANC‐1, CFPAC‐1, BxPC‐3 and SW1990 cell lines (Figure S1C). Using qRT‐PCR and western blotting, we showed that PANC‐1 had the highest levels of TBX3, followed by CFPAC‐1 cells, then SW1990 cells and lastly BxPC‐3 cells (Figure 1A,B).

### 3.2 Establishment of PDAC Cell Lines in Which TBX3 Was Stably Knocked Down

The overexpression of TBX3 in PDAC cell lines was previously reported to promote invasion, migration and angiogenesis without affecting cell proliferation, apoptosis and EMT [10]. To determine whether depleting TBX3 could inhibit these oncogenic phenotypes, we stably transfected PANC‐1 cells, which express the highest levels of TBX3, with an shRNA sequence that targets TBX3 (shTBX3). Figure 1C,D shows the successful depletion of TBX3 mRNA and protein in clones shTBX3 #1 and shTBX3 #2 that were used in this study.

### 3.3 TBX3 Knockdown Inhibited the Proliferation of PDAC Cells in 2D and 3D Culture Models

The effects of depleting TBX3 on cell proliferation were investigated using growth curve assays, and the results showed that the shTBX3 cells proliferated significantly less than shCtrl cells (Figure 1E). Light microscopy images show that while shCtrl cells had the characteristic epithelial morphology, shTBX3 cells had an enlarged and flattened morphology that is characteristic of senescent cells (Figure 1F, top). Indeed, compared to shCtrl cells, more shTBX3 cells stained blue/green with X‐gal, indicating that they have high SA‐β‐gal enzyme activity (Figure 1F, bottom). The decrease in proliferation and the senescence phenotype observed in shTBX3 cells correlated with an increase in p21^WAF1/CIP1/CDKN1A^ and a decrease in CDK2 and Cyclin B1 (Figure 1G).

To test whether depleting TBX3 inhibits PDAC growth under conditions that mimic tumours in vivo, 3D spheroids of shCtrl and shTBX3 cells were generated and their sizes were monitored over time. Figure 1H shows that depleting TBX3 significantly inhibited the growth of PDAC spheroids. Interestingly, while the shCtrl spheroids remained compact, the shTBX3 spheroids disintegrated, resulting in detached cells around their periphery. We next assessed spheroid viability using Calcein‐AM and PI, which stains for live and dead cells respectively. Fluorescence microscopy images show that while the intensity of the green Calcein‐AM stain was comparable in all spheroids, a significant increase in PI intensity was noted in the core of shTBX3 spheroids (Figure 1I,J). Furthermore, detached cells were observed in only the shTBX3 cells, and they stained green with Calcein‐AM, confirming that they were viable and suggesting that depleting TBX3 inhibited cell–cell adhesion, which may promote cell invasion and migration.

### 3.4 TBX3 Knockdown Inhibited EMT but Promoted the Migration and Invasion of PDAC Cells in 2D and 3D Culture Models

**FIGURE 2:**
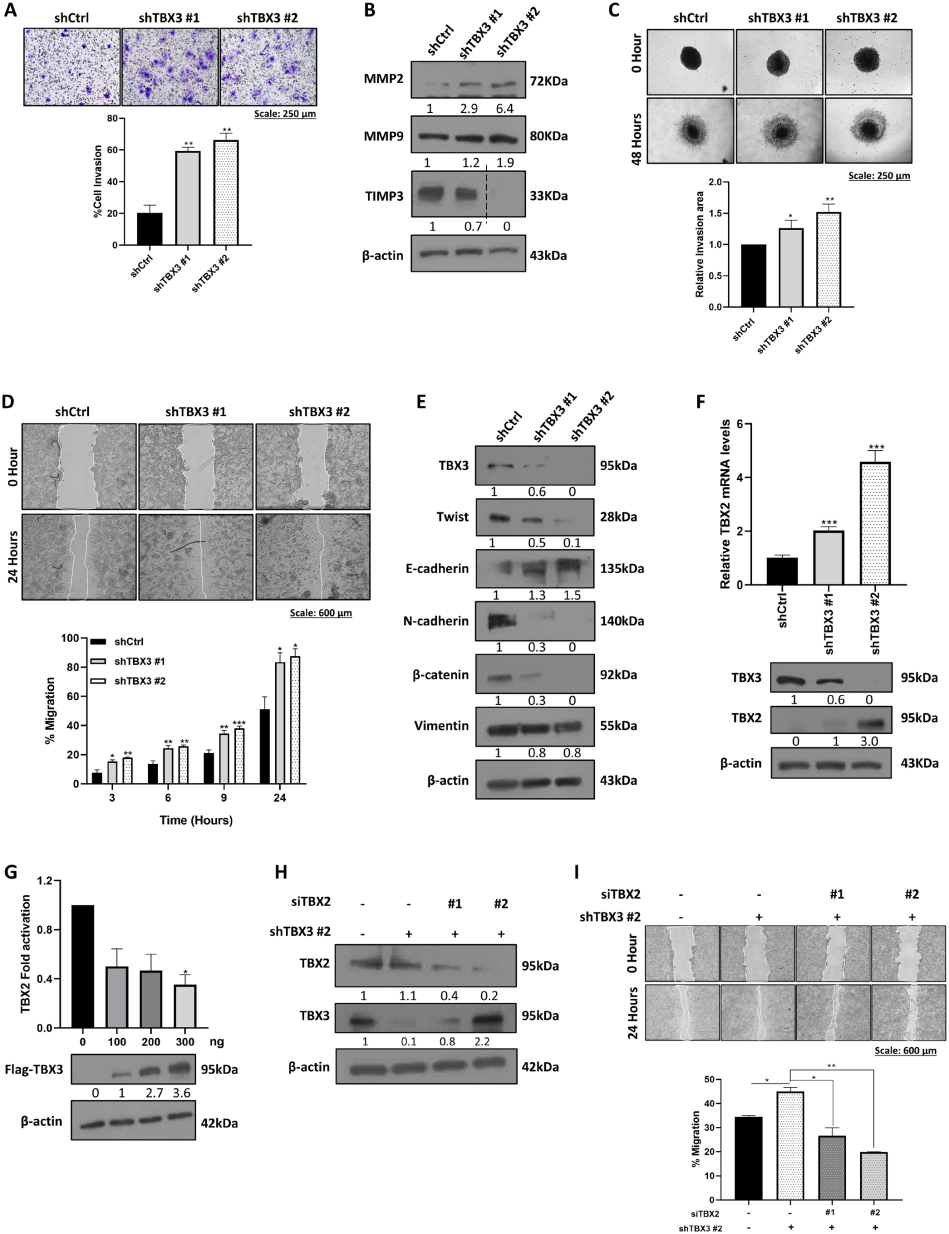
TBX3 knockdown inhibits EMT and increases levels of TBX2 to promote invasion and migration of PDAC cell cultures. (A) Representative light microscopy images (10× magnification; scale bars: 250 μm) of the transwell invasion assay of shCtrl, shTBX3 #1 and shTBX3 #2 cells. Invasive cells were stained with crystal violet, and quantified with ImageJ and the accompanying graph shows the percentage of invasive cells expressed relative to shCtrl cells. (B) Western blot analyses of cell invasion markers MMP2, MMP9 and TIMP3 in shCtrl, shTBX3 #1 and shTBX3 #2 cells. (C) Representative light microscopy images (10× magnification; scale bars: 250 μm) and quantitation of spheroid invasion assay. The shCtrl, shTBX3 #1 and shTBX3 #2 spheroids were generated, embedded in a collagen I and incubated for 48 h for the invasion to occur. (D) Representative light microscopy images (4× magnification; scale bars: 600 μm) and quantitation of the migration of shCtrl, shTBX3 #1 and shTBX3 #2 cells measured using the in vitro 2D scratch motility assay in the presence of the anti‐proliferative drug Mitomycin C. (E) Western blot analyses of EMT markers Twist, E‐cadherin, N‐cadherin, β‐catenin and vimentin in proteins harvested from the cells in (D). (F) qRT‐PCR and western blot analysis of TBX2 mRNA and protein levels in shCtrl, shTBX3 #1 and shTBX3 #2 cells. The TBX3 blot from Figure 1D was reused. (G) Luciferase reporter assays of PANC‐1 cells transfected with 200 ng of TBX2‐luciferase construct and empty vector or 100–300 ng of Flag‐TBX3. The total amount of DNA was held constant using the empty vector, and the accompanying western blot shows the expression of transfected Flag‐TBX3. (H) Western blot analyses of TBX2 in shCtrl cells and shTBX3 #2 cells transfected with siCtrl or siTBX2 #1 or siTBX2 #2. (I) Representative light microscopy images (4× magnification; scale bars: 600 μm) and quantitation of the migration of the cells in (D) measured using the in vitro 2D scratch motility assay in the presence of Mitomycin C to inhibit cell proliferation. β‐actin was used as a loading control in all western blots and data were analysed using the parametric unpaired *t*‐test by the GraphPad Prism 8.0 software. *p < 0.5, **p < 0.01, ***p < 0.0001; error bars represent mean ± SEM of three independent biological repeats.

The impact of depleting TBX3 on PDAC cell invasiveness was investigated using the transwell invasion assay. Contrary to what was previously reported [10], the results showed that shTBX3 cells were significantly more invasive than shCtrl cells (Figure 2A) and this correlated with an increase in MMP2 and MMP9 and a decrease in TIMP3 (Figure 2B). Consistent with these findings, when we assessed the ability of the shCtrl and shTBX3 spheroids to invade a collagen I matrix, which mimics the extracellular matrix, the shTBX3 spheroids were significantly more invasive than shCtrl spheroids (Figure 2C). Furthermore, in vitro 2D scratch motility assays showed that shTBX3 cells migrated significantly faster than shCtrl cells (Figure 2D). Unexpectedly, western blot analysis of proteins harvested from this assay showed that depleting TBX3 inhibited EMT, as shown by an increase in E‐cadherin and a decrease in Twist, N‐cadherin, β‐catenin and vimentin (Figure 2E).

### 3.5 Depleting TBX3 Results in Increased Levels of the TBX3 Homologue, TBX2, Which Promotes PDAC Cell Migration

The data obtained above were surprising because cell invasion and migration are preceded by EMT and therefore, inhibiting EMT should block these processes. This is, however, consistent with a study that reported that deleting *Snail* or *Twist* in PDAC mouse models did not affect the emergence of invasive PDAC, systemic dissemination or metastasis [27]. Interestingly, the expression of TBX2, which is highly homologous to TBX3, correlates directly with late TNM stage and distant metastasis in PDAC and there is evidence that TBX3 can transcriptionally repress TBX2 [28, 29, 30, 31]. We therefore hypothesised that depleting TBX3 results in the upregulation of TBX2 which then promoted the invasion and migration of shTBX3 PDAC cells. Indeed, our results show that depleting TBX3 led to an increase in TBX2 mRNA and protein (Figure 2F) and luciferase assays revealed that this was due to a loss of transcriptional repression of *TBX2* by TBX3 (Figure 2G). We next depleted TBX2 in shTBX3 cells (Figure 2H) and measured their migratory ability. As previously shown, shTBX3 cells migrated faster than shCtrl cells, however, when TBX2 was depleted in shTBX3 cells, they migrated significantly less than shCtrl cells (Figure 2I). Together, the data revealed that depleting TBX3 increased the levels of TBX2 which promoted the migration of PDAC cells, and that an effective PDAC drug needs to inhibit both TBX2 and TBX3.

### 3.6 The Cytotoxic Effects of Piroctone Olamine (PO) in PDAC Cells and Spheroids as Well as PDAC Patient‐Derived Organoids

**FIGURE 3:**
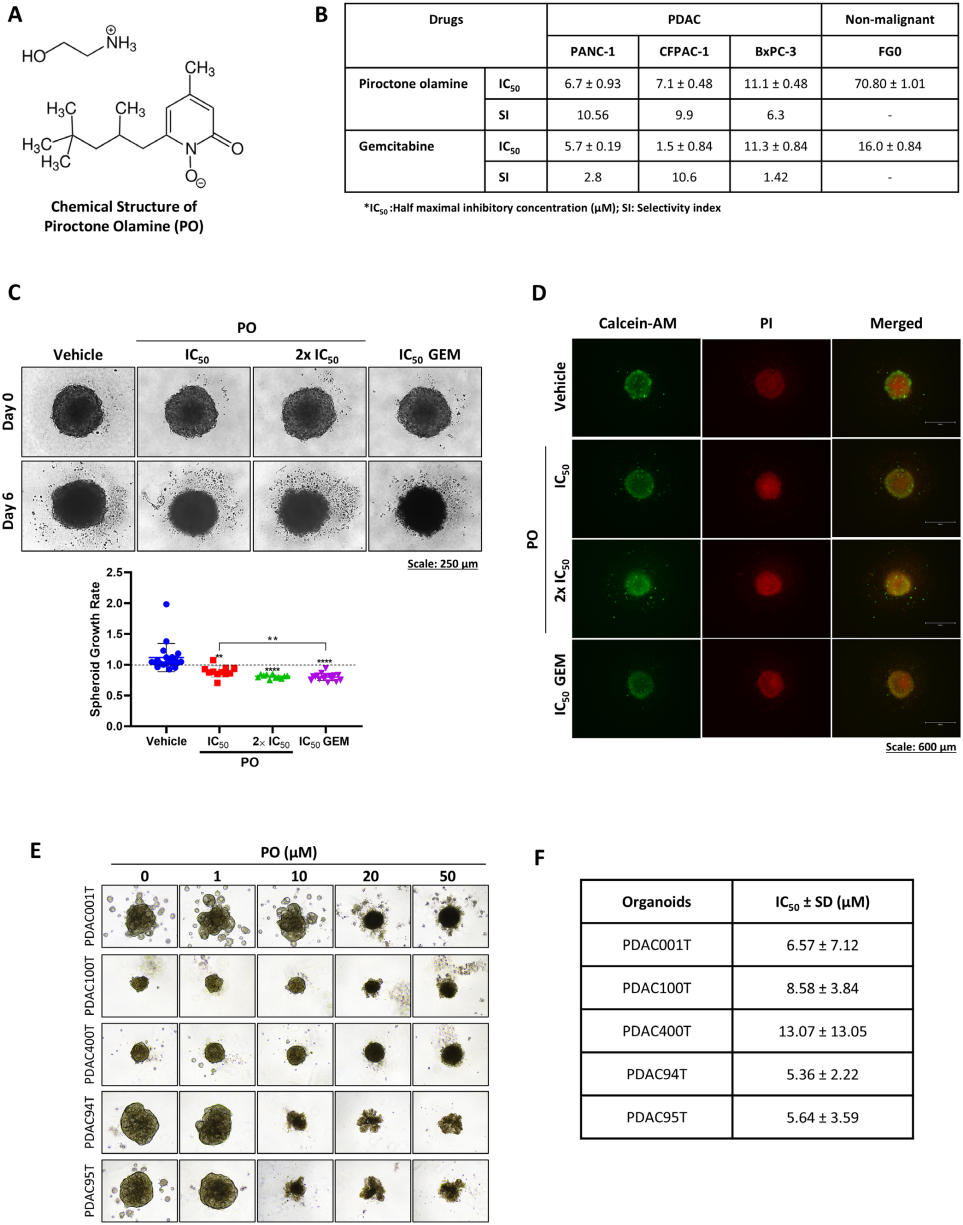
The cytotoxic effects of piroctone olamine in PDAC cells, spheroids and patient‐derived organoids. (A) Chemical structure of piroctone olamine (PO). (B) The half maximal inhibitory concentrations (IC_50_) determined from MTT cell viability assays of PANC‐1, CFPAC‐1 and BxPC‐3 PDAC cell lines and non‐malignant FG0 skin fibroblasts post 72 h of treatment with increasing concentrations of PO and gemcitabine (GEM). Selectivity indices (SI) were calculated by dividing the IC_50_ FG0 cells by the IC_50_ of each PDAC cell line. (C) Representative light microscope images (10× magnification; scale bars: 250 μm) of PDAC spheroids treated for 6 days as indicated. The spheroid area was measured using the ImageJ software, and the spheroid growth rate was calculated by dividing the spheroid area on day 6 by its respective area on day 0. (D) Representative fluorescence microscope images (4× magnification; scale bars: 600 μm) of the spheroids in (C) stained with Calcein‐AM (live cells; green), propidium iodide (dead cells; red) and DAPI on day 6. (E) Representative light microscope images (10× magnification; scale bars: 250 μm) of PDAC patient‐derived organoids treated for 6 days with increasing concentrations of PO. (F) IC_50_ values show PO's effect on the organoids' viability. The viability of the organoids was measured using the CellTiter‐Glo 3D Cell Viability Assay and IC_50_ values were interpolated from the dose response curves using the GraphPad Prism 8.0 software. **p < 0.01, ****p < 0.0001; error bars represent mean ± SEM of three independent biological repeats.

PO (Figure 3A) was previously identified as a TBX2‐ and TBX3‐targeting drug in TBX2 and TBX3 dependent melanoma and rhabdomyosarcoma cells [16]. It was therefore hypothesised that PO will also inhibit TBX2 and TBX3 levels in PDAC cells and recapitulate the phenotype observed when they were both depleted in PDAC cells. To begin to investigate this, PANC‐1, CFPAC‐1 and BxPC‐3 cells were treated with PO and MTT assays were performed to calculate IC_50_ values in these cells. In these experiments, GEM was included as a positive control drug and to determine the selectivity index (SI) of PO, the non‐malignant FG0 cells were included. Figure 3B shows that for PO, IC_50_ values of 6.7, 7.1 and 11.1 μM were obtained in PANC‐1, CFPAC‐1 and BxPC3 cells and GEM had IC_50_ values of 5.7, 1.5 and 11.3 μM in the same cell lines respectively. Furthermore, unlike GEM, PO was highly selective for all PDAC cell lines tested as indicated by SI values > 5 (Figure 3B). Importantly, PO significantly reduced the size and viability of PDAC spheroids (Figure 3C,D) and PDAC patient‐derived organoids (PDOs) with IC_50_ values ranging from 4.5 to 13.1 μM in all PDOs tested (Figure 3E,F). Collectively, these findings highlighted the strong cytotoxic potential and promise of PO as a novel therapeutic agent for PDAC.

### 3.7 Piroctone Olamine Inhibits Long‐Term Survival, Migration and Invasion of PDAC Cells and Spheroids

**FIGURE 4:**
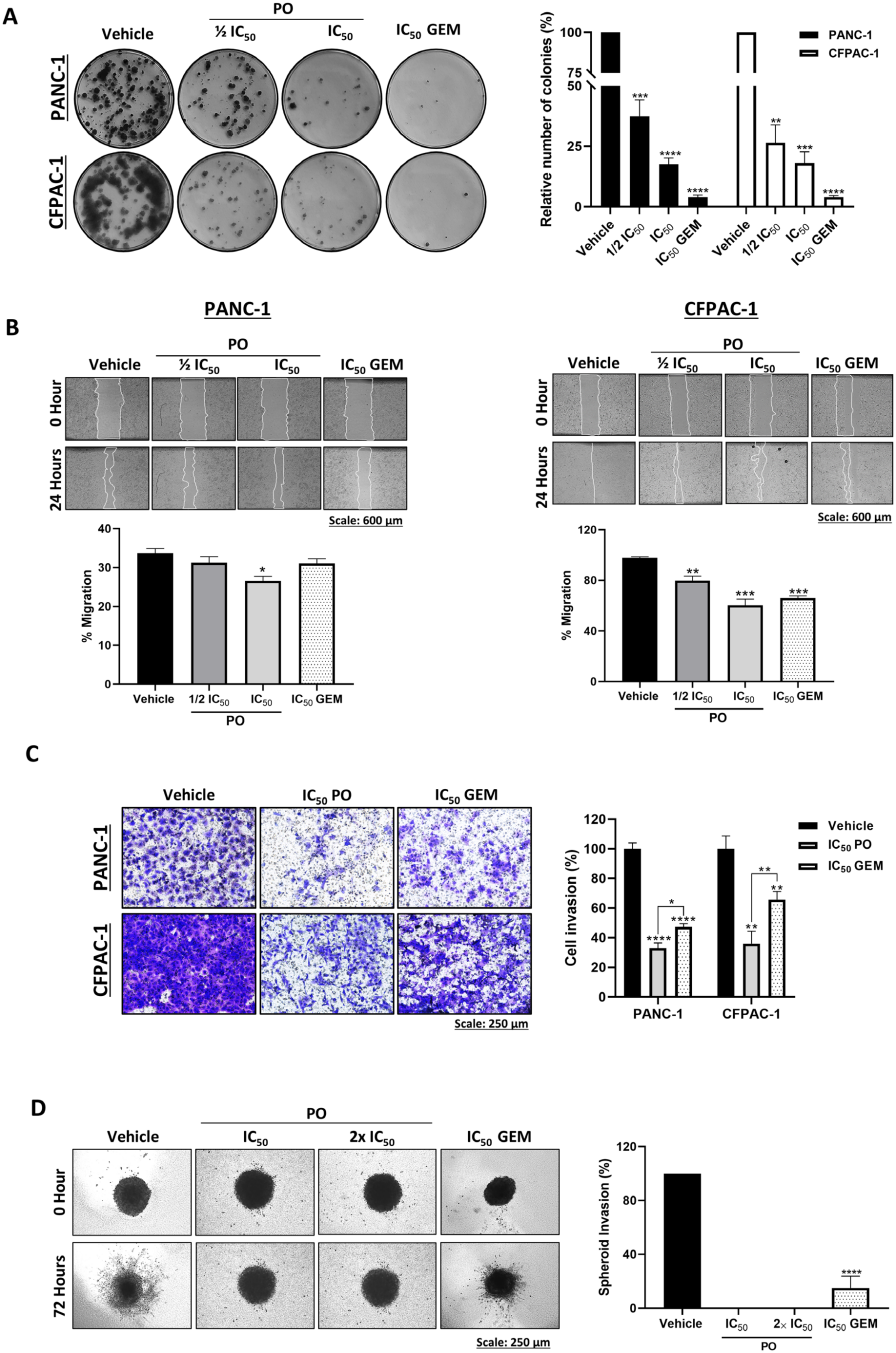
Piroctone olamine inhibits long‐term survival, migration and invasion of PDAC cells and spheroids. (A) Representative images and quantitation of the clonogenic assays of PANC‐1 and CFPAC‐1 cells. Colonies were stained with crystal violet, imaged and quantified using the ColonyArea plugin of the ImageJ software. (B) Representative light microscopy images (4× magnification; scale bars: 600 μm) and quantitation of the migration of PANC‐1 and CFPAC‐1 cells in the presence of the indicated concentrations of PO or GEM. Cell migration was measured using the in vitro 2D scratch motility assay and Mitomycin C was used to inhibit cell proliferation. (C) Representative light microscopy images (10× magnification; scale bars: 250 μm) and quantitation of transwell invasion assays of PO‐ or GEM‐treated PANC‐1 and CFPAC‐1 cells. Invasive cells were stained with crystal violet and quantified using the ImageJ software. (D) Representative light microscopy images (10× magnification; scale bars: 250 μm) and quantitation of the invasion of the PDAC spheroids that were established, embedded into collagen I and treated for 72 h as indicated. Data were analysed using the parametric unpaired *t*‐test by the GraphPad Prism 8.0 software. *p < 0.5, **p < 0.01, ***p < 0.001, ****p < 0.0001; error bars represent mean ± SEM of three independent biological repeats.

Clonogenic assays showed that PO inhibited the long‐term survival of PANC‐1 and CFPAC‐1 cells because it significantly inhibited their colony‐forming ability, albeit to a lesser extent than GEM (Figure 4A). Furthermore, as shown in Figure 4B,C, PO significantly inhibited the migration and invasion ability of PANC‐1 and CFPAC‐1, and it was significantly more effective than GEM. Consistent with these findings, PO significantly inhibited the invasion of PDAC spheroids, as indicated by the absence of spindles which were observed in vehicle‐ and GEM‐treated spheroids (Figure 4D). Together, the results showed that PO was more effective than GEM at inhibiting the invasion and migration of PDAC cells and 3D spheroids.

### 3.8 Piroctone Olamine Induces DNA Damage, Cell Cycle Arrests and Senescence in PDAC Cells

**FIGURE 5:**
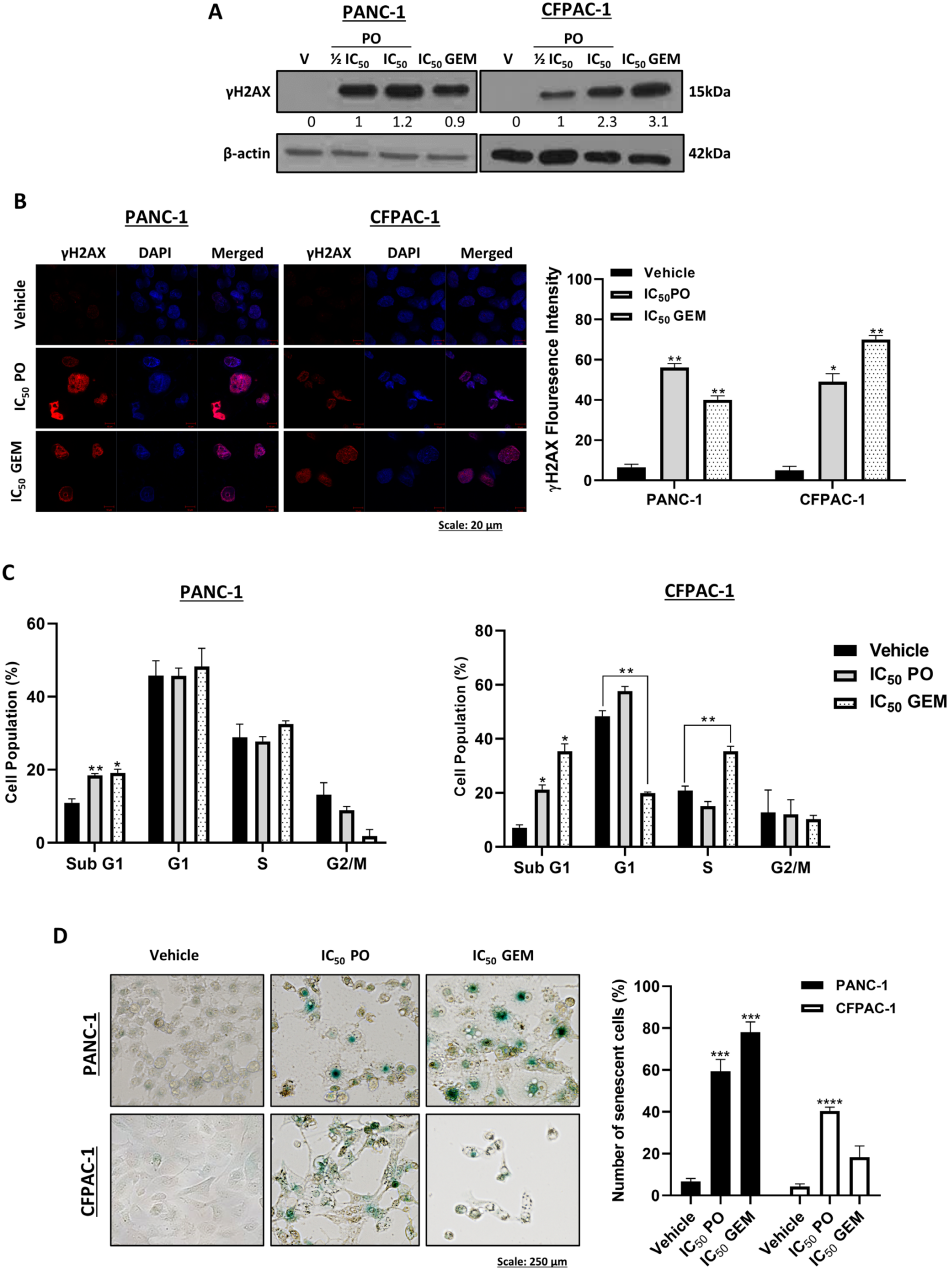
Piroctone olamine induces double‐strand DNA breaks, cell cycle arrests and senescence in PDAC cells. (A) Western blot analysis of γH2AX levels in PANC‐1 and CFPAC‐1 cells treated for 72 h as indicated. β‐actin was used as a loading control, and densitometric readings were expressed relative to the first lane with a band. (B) Representative confocal immunofluorescence images (630× magnification; scale bars: 20 μM) and quantitation of ƴH2AX foci in PANC‐1 and CFPAC‐1 cells treated as indicated. ƴH2AX was detected with a fluorophore conjugated Cy3 secondary antibody and nuclei were stained with DAPI. (C) Flow cytometry analysis of PO‐ or GEM‐treated PANC‐1 and CFPAC‐1 cells stained with propidium iodide for cell cycle distribution. The graph represents the mean proportion of cells in each phase of the cell cycle. (D) Representative brightfield microscopy images (10× magnification; scale bars: 250 μm) and quantitation of the senescence‐associated β‐galactosidase staining of PANC‐1 and CFPAC‐1 cells treated as indicated. Cells were imaged from 15 fields of view, and senescent cells were counted, and data were expressed as a percentage of vehicle‐treated cells. Data were analysed using the parametric unpaired *t*‐test by the GraphPad Prism 8.0 software. *p < 0.5, **p < 0.01, ***p < 0.001, ****p < 0.0001; error bars represent mean ± SEM of three independent biological repeats.

To investigate the mechanisms by which PO exerts its cytotoxicity, we first investigated whether it induces DNA damage by measuring γH2AX levels by western blotting and immunocytochemistry. Figure 5A,B shows that PO increased γH2AX levels and nuclei puncta in PANC‐1 and CFPAC‐1 cells. Flow cytometry analyses revealed that there was a comparable number of PANC‐1 cells in G1 and S phase across all treatments but, albeit not significant, a decrease in the number of G2/M cells treated with PO and GEM (Figure 5C). In CFPAC‐1 cells, however, PO treatment resulted in a G1 arrest and GEM treatment in an S‐phase arrest (Figure 5C). Importantly, PO treatment resulted in a significant increase in the number of PDAC cells in sub‐G1 (Figure 5C) which is indicative of DNA fragmentation and cell death [32]. In addition, the SA‐β‐gal staining assay revealed that PO induced senescence in both PDAC cell lines, but GEM did so only in PANC‐1 cells (Figure 5D).

### 3.9 Piroctone Olamine Induces Apoptosis and Autophagic Flux in PDAC

**FIGURE 6:**
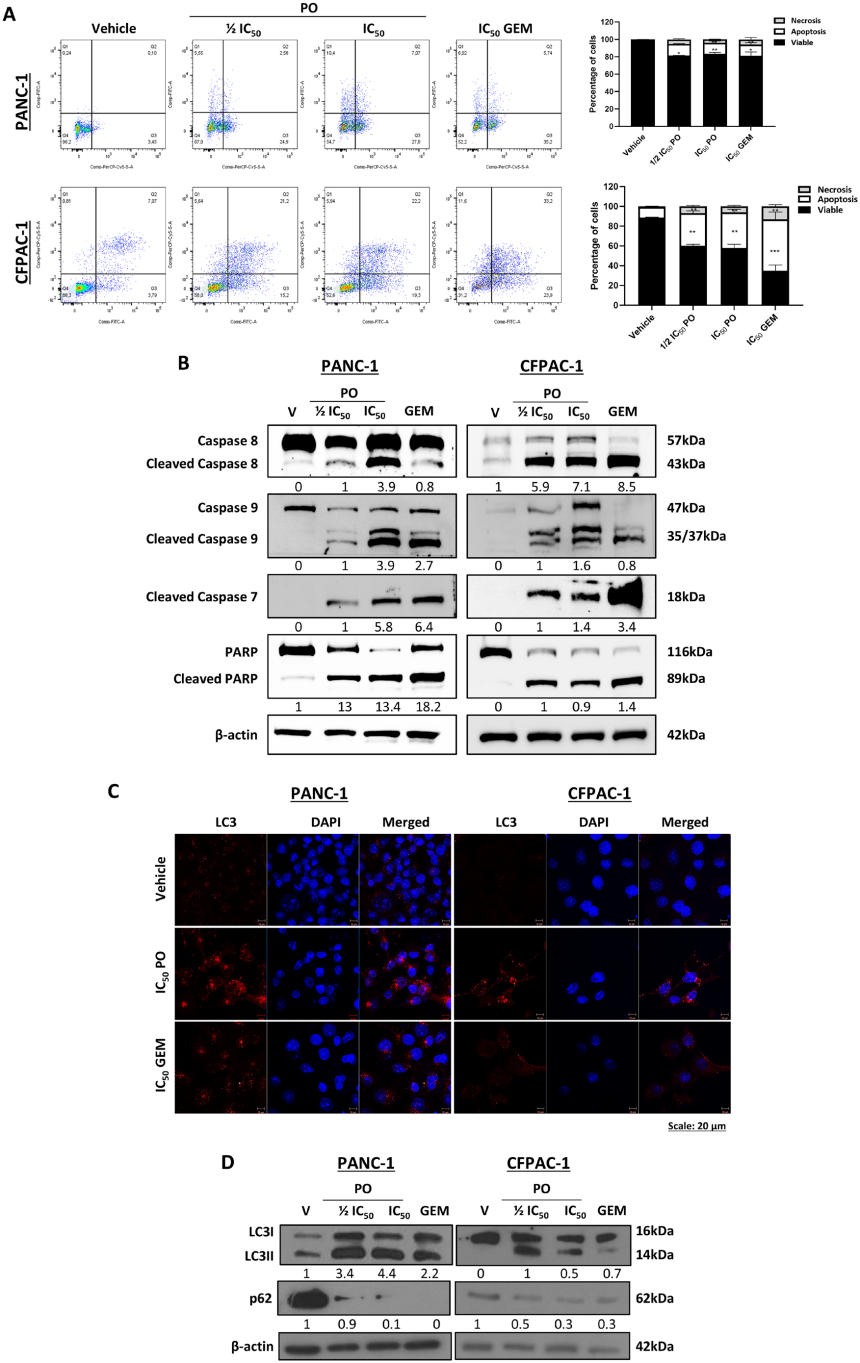
Piroctone olamine induces apoptosis and autophagic flux in PDAC cells. (A) Annexin V‐FITC/PI flow cytometry analysis and quantitation of viable, apoptotic (early and late apoptosis) and necrotic cells after treatment with PO or GEM as indicated. Lower left‐hand quadrant: viable cells; lower right‐hand + upper right‐hand quadrants: Apoptotic cells and upper left‐hand quadrant: necrotic cells. (B) Western blot analyses of apoptosis markers caspase‐8, caspase‐9, cleaved caspase‐7 and PARP in PANC‐1 and CFPAC‐1 cells treated as indicated. (C) Representative confocal immunofluorescence images (400× magnification, scale bar: 20 μM) of LC3 in PANC‐1 and CFPAC‐1 cells treated as indicated. LC3 was detected with a fluorophore conjugated Cy3 secondary antibody and nuclei were stained with DAPI. (D) Western blot analyses of the autophagy markers LC3 and p62 in cells treated as indicated. For all western blots, β‐actin was used as a loading control, and densitometric readings were expressed relative to the vehicle‐treated control or the first lane with a band. Data were analysed using the parametric unpaired *t*‐test by the GraphPad Prism 8.0 software. *p < 0.05, **p < 0.01, ***p < 0.001; error bars represent mean ± SEM of three independent biological repeats.

To explore whether PO induced PDAC cell death, Annexin V‐FITC/PI and flow cytometry analysis were performed. Figure 6A shows that PO induced a significant increase in total apoptosis in both PDAC cell lines, which was comparable to GEM in PANC‐1 cells but less than GEM in CFPAC‐1 cells. Western blotting revealed that PO triggered both intrinsic and extrinsic apoptosis, as shown by increased levels of cleaved caspases‐ 8, 9, 7 and PARP (Figure 6B). We further show that PO and GEM induced autophagic flux in both PDAC cell lines, since they increased LC3 puncta (Figure 6C) and levels of LC3II, and decreased levels of p62 (Figure 6D).

### 3.10 Piroctone Olamine Inhibits TBX2 and TBX3 Levels and Exerts Its Cytotoxicity by Targeting TBX3 in PDAC Cells

**FIGURE 7:**
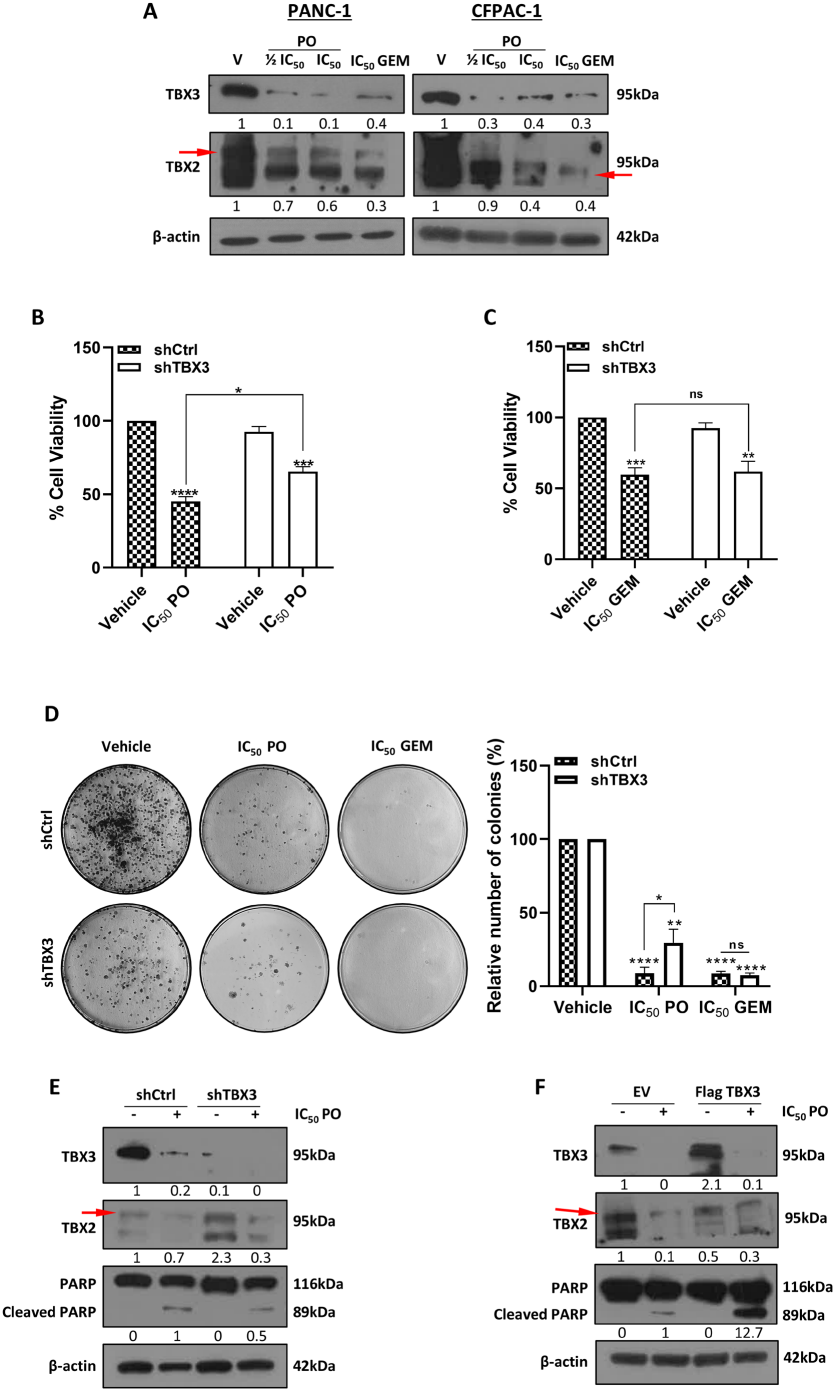
The cytotoxic and pro‐apoptotic effects of piroctone olamine depend on TBX3 levels. (A) Western blot analyses of TBX2 and TBX3 levels in PANC‐1 and CFPAC‐1 cells post 72 h of treatment as indicated. The red arrows indicate the TBX2 band. (B, C) MTT cell viability assays of PANC‐1 shCtrl and shTBX3 cells treated for 72 h as with IC_50_ PO (B) or GEM (C). (D) Representative images and quantitation of clonogenic assays of PANC‐1 shCtrl and shTBX3 cells treated as indicated. Colonies were stained with crystal violet and quantified using the ColonyArea plugin of the ImageJ software. (E) Western blot analyses of TBX2, TBX3 and PARP in shCtrl and shTBX3 cells treated as in (B). (F) Western blot analyses of TBX2, TBX3 and PARP in PANC‐1 cells transfected with pCMV Flag‐empty (EV) or pCMV Flag‐TBX3 and treated with as in (B). β‐actin was used as a loading control in all western blots, and densitometric readings were expressed relative to the vehicle‐treated shCtrl or EV cells for TBX3 and IC_50_ PO‐treated shCtrl or EV cells (i.e., first lanes with a band) for cleaved PARP. Data were analysed using the parametric unpaired *t*‐test by the GraphPad Prism 8.0 software. ns, not significant, *p < 0.5, **p < 0.01, ***p < 0.001, ****p < 0.001; error bars represent mean ± SEM of three independent biological repeats.

As previously mentioned, an effective anti‐PDAC drug should inhibit both TBX2 and TBX3 levels, and we therefore next investigated the effects of PO on these two proteins in PANC‐1 and CFPAC‐1 cells. Figure 7A shows that PO reduced TBX2 and TBX3 levels, and interestingly, GEM also inhibited the levels of these proteins. These results suggest that treatment with PO may recapitulate the phenotypes observed when TBX2 and TBX3 were depleted in PDAC cells. To test this, we determined whether the inhibition of PANC‐1 proliferation and colony forming ability by PO required, in part, its ability to inhibit TBX3. Indeed, MTT and clonogenic assays showed that, compared to shCtrl cells, PO was significantly less effective at inhibiting the short‐term viability and long‐term survival of shTBX3 cells (Figure 7B−D). Interestingly, in these experiments, there were no significant differences between the sensitivity of shCtrl cells and shTBX3 cells to GEM (Figure 7C,D). We next determined whether PO required TBX3 to induce apoptosis by measuring the levels of cleaved PARP in PANC‐1 cells in which TBX3 was either depleted or overexpressed. The results showed that PO treatment resulted in lower levels of cleaved PARP in shTBX3 cells compared to shCtrl cells (Figure 7E) and in higher levels of cleaved PARP in Flag‐TBX3 cells compared to EV cells (Figure 7F). Collectively, these data showed that the effectiveness of PO to induce cytotoxicity and apoptotic cell death in PDAC cells is, in part, dependent on its ability to inhibit TBX3 levels.

## 4 Discussion

PDAC remains intractable and has a 5‐year survival rate of < 10% [2]. Efforts to target key drivers of PDAC have been marginally successful and, therefore, considerable progress is required to identify and validate novel molecular targets for the development of PDAC therapies [3, 5]. TBX3 is overexpressed in multiple cancers, and it has been shown to contribute to several oncogenic processes and has been validated as a therapeutic target [7]. In PDAC, TBX3 was shown to promote invasion, migration and angiogenesis [10], but to date, the impact of biologically inhibiting TBX3 on these processes has not been investigated. Here, we show that depleting TBX3 in PDAC cells induces senescence and inhibits proliferation, EMT and spheroid growth. Furthermore, we reveal that TBX3 transcriptionally represses TBX2 and that when TBX3 is depleted, TBX2 levels increase and result in enhanced PDAC cell migration. These results thus demonstrate that TBX2 and TBX3 have distinct oncogenic functions in PDAC and that any effective anti‐PDAC drug must inhibit them both. With this in mind, we explored the potential of PO, which was previously shown to inhibit TBX2 and TBX3 in TBX2‐/3‐dependant melanoma and rhabdomyosarcoma cells, to inhibit levels of these two proteins in PDAC cells. Indeed, we demonstrate that in PDAC cells, PO inhibited TBX2 and TBX3 levels and exhibited potent cytotoxicity in 2D cell culture and 3D spheroids as well as PDAC patient‐derived organoids and recapitulated the anti‐cancer activities observed when TBX2 and TBX3 were depleted in 2D and 3D PDAC cell culture models.

Our findings are consistent with previous reports that TBX3 promotes cancer cell survival and proliferation by functioning as an anti‐senescence factor and repressing the levels of p16^INK4a^, pRb and p21^WAF1/CIP1^ [7, 33, 34]. Interestingly, these data are opposite to that obtained in a study by Perkhofer et al. which showed that the ectopic expression of TBX3 in PANC‐1 and BxPC3 PDAC cells promoted their migration, invasion and angiogenesis but had no impact on cell proliferation, apoptosis and EMT [10]. This may be due to Perkhofer et al. using a TBX3 overexpression model and our study using a knockdown model in which only TBX3 was depleted. Indeed, gene overexpression may result in non‐physiological levels of a protein which leads to artificial phenotypes, and Wang et al. showed that ectopically overexpressing a protein can favour one protein subunit or isoform over the other(s) [35, 36]. It is worth noting that there are two predominant TBX3 isoforms, namely, TBX3 and TBX3 + 2a, which can exhibit opposite functions depending on cellular contexts [7, 36]. For example, while TBX3 was found to bind to a consensus T‐box‐binding site to inhibit senescence in mouse embryonic fibroblasts, TBX3 + 2a was unable to bind this site and induce senescence [37]. It is, therefore, possible that TBX3 and TBX3 + 2a contribute to different aspects of PDAC and that the lentiviral TBX3 overexpression construct used in the Perkhofer et al. (2016) study may have favoured the expression of the TBX3 isoform that promotes the phenotype they observed. Importantly, knockdown models mimic loss of function mutations and provide an accurate account of the impact of reducing the expression of a specific gene on cellular functions [38]. Indeed, the shRNA sequence used in this study targets both TBX3 isoforms, and therefore, we are confident that the phenotypes we observed occurred due to the loss of function of both TBX3 and TBX3 + 2a. To resolve the different results obtained in our study and that of Perkhofer et al. future studies should identify which of the two TBX3 isoforms is responsible for the PDAC phenotype observed by Perkhofer et al. by assessing the effects of overexpressing each isoform in PDAC cells.

The current study showed that depleting TBX3 in PDAC cells increased their migration and invasion but inhibited EMT. This was a surprising finding since it is well documented that for cancer cells to acquire the ability to invade and migrate, they need to undergo EMT [27]. There is, however, evidence that cancer cell invasion and migration can occur independently of EMT. For example, Schaeffer et al. demonstrated that EMT was not a pre‐requisite for mammary carcinoma cells to acquire a migratory phenotype, and Schelch et al. showed that in lung adenocarcinoma cells, epidermal growth factor induced cell migration independent of EMT [39, 40]. Furthermore, Zheng et al. demonstrated using PDAC mouse models that inhibiting EMT by genetic deletion of *Snail* or *Twist1* did not affect the emergence of invasive PDAC, systemic PDAC dissemination or PDAC metastasis [41]. Our results therefore add to the body of work that shows that EMT is not always required for cancer cell invasion and migration.

TBX3, and its homologue TBX2, were both reported to be highly expressed in PDAC patient tissues, which correlated directly with late TNM stage and distant metastasis, and 50% of PDAC cell lines tested were shown to express high levels of TBX2 [9, 28, 29, 42]. Furthermore, TBX2 and TBX3 play distinct but causative oncogenic roles in cancers where they are both expressed, and they repress one another to achieve these functions [21, 30, 31]. For example, in breast cancer and melanoma, TBX2 functions as a powerful pro‐proliferative factor while TBX3 promotes invasion and migration by repressing TBX2 [21]. However, contrary to these findings, our data showed that in PDAC cells, TBX3 functions as an anti‐senescence and pro‐proliferative factor, while TBX2 promotes cell migration. Together, our findings highlighted the functional significance of TBX2 and TBX3 in PDAC cells and suggested that targeting TBX2 and TBX3 concurrently may be a valuable strategy for effective PDAC treatment.

PO reduced the levels of TBX2 and TBX3 and exerted its cytotoxicity, in part, through its ability to inhibit TBX3 levels. Future studies will need to confirm if PO inhibited PDAC cell invasion and migration by targeting TBX2 and determine whether PO reduces TBX2 and TBX3 levels through transcriptional or post‐translational mechanisms. This could be assessed using qRT‐PCR to measure mRNA levels and by combining PO treatment with a proteasome inhibitor such as MG132 to measure protein stability. It is worth noting that TBX2 and TBX3 have been implicated in conferring tumour drug resistance, and because PO can target them, it may be associated with reduced tumour drug resistance. Indeed, TBX2 confers resistance to temozolomide in glioblastoma, cisplatin in breast cancer and melanoma and platinum‐based chemotherapeutics in ovarian serous carcinoma [10, 43]. TBX3, on the other hand, confers tumour drug resistance by bypassing anoikis and promoting the expansion and maintenance of breast and PDAC cancer stem cells [44, 45, 46, 47]. Furthermore, TBX3 functions as an anti‐senescence factor and, therefore, the pro‐senescence effects of PO may be attributed to its ability to inhibit TBX3 levels [7]. Senescent cells can upregulate anti‐apoptotic pathways to bypass cell death despite the accumulation of DNA damage, thus leading to therapy resistance [48]. It would therefore be interesting to investigate the effects of treating PDAC cells with a combination of PO and senolytics, a class of drugs that function by selectively targeting senescent cells [49]. One example would be Navitoclax (ABT‐263), which exerts its senolytic activity by targeting Bcl‐2 anti‐apoptotic proteins, and when administered in combination with gemcitabine, it was demonstrated to kill gemcitabine‐resistant and senescent‐like pancreatic cancer cells [50].

## 5 Conclusions

Our study revealed that TBX3 promoted PDAC cell proliferation and EMT and highlighted the functional involvement of TBX2 in PDAC cell invasion and migration. We demonstrated that in PDAC cells, PO inhibited the levels of TBX2 and TBX3 and phenocopied the depletion of TBX2 and TBX3 in these cells. Furthermore, PO exerted its cytotoxicity, in part, through its ability to target TBX3. We propose that PO is a potential drug candidate to be repurposed for the treatment of TBX2‐/3‐dependent PDAC.

## Author Contributions

**Karabo Serala:** formal analysis, investigation, validation and data curation, writing original draft, review and editing. **Sanele Mdletshe:** formal analysis, investigation, validation and data curation. **Jinming Bai:** formal analysis, investigation, validation and data curation. **Amaal Abrahams:** supervision and writing, review and editing. **Odile Gayet:** supervision, formal analysis, investigation, validation and data curation. **Loic Moubri:** supervision, formal analysis, investigation, validation and data curation. **Nelson Dusetti:** resources, supervision, formal analysis, investigation, validation and data curation. **Sharon Prince:** conceptualisation, formal analysis, resources, writing original draft, review and editing, supervision, project administration and funding acquisition.

## Ethics Statement

PDAC samples used for organoid derivation were obtained from three expert clinical centres under the PaCaOmics clinical trial (number 2011‐A01439‐32) after receiving ethics review board approval.

## Consent

Consent forms of informed patients were collected.

## Conflicts of Interest

The authors declare no conflicts of interest.

## Supporting information


**Table S1.** Western blotting antibody list.
**Table S2.** Immunofluorescence antibody list.

**Figure S1.** The expression of TBX3 and association with patient survival in PDAC. (A) Analysis of The Cancer Genome Atlas PDAC cohort (TCGA‐PAAD) database for *TBX3* levels in PDAC patient tissues and adjacent normal tissues. (B) Kaplan–Meier analysis of the survival rates between PDAC tumours that express high *TBX3* and those that express low *TBX3*. (C) Analysis of the Cancer Cell Line Encyclopaedia (CCLE) database for *TBX3* levels in PDAC cell lines (*n = 46*).

## Data Availability

The data presented in this article are available.

## References

[1] H. Sung , J. Ferlay , R. L. Siegel , et al., “Global Cancer Statistics 2020: GLOBOCAN Estimates of Incidence and Mortality Worldwide for 36 Cancers in 185 Countries,” CA: A Cancer Journal for Clinicians 71, no. 3 (2021): 209–249, https://onlinelibrary.wiley.com/doi/10.3322/caac.21660. 33538338 10.3322/caac.21660

[2] J. Natu and G. P. Nagaraju , “Gemcitabine Effects on Tumor Microenvironment of Pancreatic Cancer: Special Focus on Resistance Mechanisms and Metronomic Therapies,” Cancer Letters 573 (2023): 216382, https://linkinghub.elsevier.com/retrieve/pii/S0304383523003336. 37666293 10.1016/j.canlet.2023.216382

[3] A. Turpin , C. Neuzillet , E. Colle , et al., “Therapeutic Advances in Metastatic Pancreatic Cancer: A Focus on Targeted Therapies,” Therapeutic Advances in Medical Oncology 14 (2022): 175883592211180, http://journals.sagepub.com/doi/10.1177/17588359221118019. 10.1177/17588359221118019 PMC9459481 36090800

[4] C. J. Halbrook , C. A. Lyssiotis , M. Pasca di Magliano , and A. Maitra , “Pancreatic Cancer: Advances and Challenges,” Cell 186, no. 8 (2023): 1729–1754, https://linkinghub.elsevier.com/retrieve/pii/S0092867423001423. 37059070 10.1016/j.cell.2023.02.014 PMC10182830

[5] J. H. Strickler , H. Satake , T. J. George , et al., “Sotorasib in KRAS p.G12C–Mutated Advanced Pancreatic Cancer,” New England Journal of Medicine 388, no. 1 (2023): 33–43, http://www.nejm.org/doi/10.1056/NEJMoa2208470. 36546651 10.1056/NEJMoa2208470 PMC10506456

[6] H. S. Walter and S. I. Ahmed , “Targeted Therapies in Cancer,” Surgery 39, no. 4 (2021): 202–207, https://linkinghub.elsevier.com/retrieve/pii/S0263931921000259.

[7] S. F. Khan , V. Damerell , R. Omar , et al., “The Roles and Regulation of TBX3 in Development and Disease,” Gene 726 (2020): 144223, https://linkinghub.elsevier.com/retrieve/pii/S0378111919308820. 31669645 10.1016/j.gene.2019.144223 PMC7108957

[8] S. Begum and V. E. Papaioannou , “Dynamic Expression of Tbx2 and Tbx3 in Developing Mouse Pancreas,” Gene Expression Patterns 11, no. 8 (2011): 476–483, http://www.ncbi.nlm.nih.gov/pubmed/21867776. 21867776 10.1016/j.gep.2011.08.003 PMC3200443

[9] H.‐C. Wang , Q.‐C. Meng , Z.‐Z. Shan , Z. Yuan , and X.‐Y. Huang , “Overexpression of Tbx3 Predicts Poor Prognosis of Patients With Resectable Pancreatic Carcinoma,” Asian Pacific Journal of Cancer Prevention 16, no. 4 (2015): 1397–1401, http://koreascience.or.kr/journal/view.jsp?kj=POCPA9&py=2015&vnc=v16n4&sp=1397. 25743805 10.7314/apjcp.2015.16.4.1397

[10] L. Perkhofer , K. Walter , I. G. Costa , et al., “Tbx3 Fosters Pancreatic Cancer Growth by Increased Angiogenesis and Activin/Nodal‐Dependent Induction of Stemness,” Stem Cell Research 17, no. 2 (2016): 367–378, https://linkinghub.elsevier.com/retrieve/pii/S1873506116301064. 27632063 10.1016/j.scr.2016.08.007

[11] M. Shakibaie , M. Haghiri , M. Jafari , et al., “Preparation and Evaluation of the Effect of Fe_3_O_4_ @Piroctone Olamine Magnetic Nanoparticles on Matrix Metalloproteinase‐2: A Preliminary In Vitro Study,” Biotechnology and Applied Biochemistry 61, no. 6 (2014): 676–682. 24716879 10.1002/bab.1231

[12] A. E. Albalawi , A. K. Khalaf , M. S. Alyousif , et al., “Fe_3_O_4_@Piroctone Olamine Magnetic Nanoparticles: Synthesize and Therapeutic Potential in Cutaneous Leishmaniasis,” Biomedicine & Pharmacotherapy 139 (2021): 111566. 33839494 10.1016/j.biopha.2021.111566

[13] F. M. do Couto , S. C. do Nascimento , S. F. Júnior , V. K. da Silva , A. F. Leal , and R. P. Neves , “Antifungal Activity of the Piroctone Olamine in Experimental Intra‐Abdominal Candidiasis,” Springerplus 5, no. 1 (2016): 468. 27119072 10.1186/s40064-016-2130-8 PMC4833761

[14] Y. Kim , P. Alpmann , S. Blaum‐Feder , et al., “Increased In Vivo Efficacy of Lenalidomide by Addition of Piroctone Olamine,” In Vivo 25, no. 1 (2011): 99–103. 21282741

[15] W. Xu , J. Ye , F. Wang , and T. Chen , “Piroctone Olamine Disrupts Mitochondrial Dynamics in Glioma Cells Through the PI3K/AKT Pathway,” Nan Fang Yi Ke Da Xue Xue Bao 43, no. 5 (2023): 764–771, http://www.ncbi.nlm.nih.gov/pubmed/37313818. 37313818 10.12122/j.issn.1673-4254.2023.05.12 PMC10267238

[16] J. S. Bleloch , S. Lu , S. F. Khan , et al., “A High‐Throughput Drug Repurposing Strategy to Treat TBX2 and/or TBX3 Dependent Cancers,” Cancer Medicine 13, no. 19 (2024): e70303. 39403898 10.1002/cam4.70303 PMC11474296

[17] C. S. Messina , H. Weiher , and I. G. H. Schmidt‐Wolf , “Targeting Prostate Cancer With a Combination of WNT Inhibitors and a Bi‐Functional Peptide,” Anticancer Research 37, no. 2 (2017): 555–559. 28179301 10.21873/anticanres.11348

[18] S. A. Von Schulz‐Hausmann , L. C. Schmeel , F. C. Schmeel , and I. G. H. Schmidt‐Wolf , “Targeting the Wnt/Beta‐Catenin Pathway in Renal Cell Carcinoma,” Anticancer Research 34, no. 8 (2014): 4101–4108. 25075035

[19] I. Wall and I. G. H. Schmidt‐Wolf , “Effect of Wnt Inhibitors in Pancreatic Cancer,” Anticancer Research 34, no. 10 (2014): 5375–5380. 25275031

[20] K. Serala , J. Bai , and S. Prince , “Pyrvinium Pamoate Alone and With Gemcitabine Exhibits Anti‐Pancreatic Cancer Activity in 2D and 3D Cell Culture Models,” Journal of Cellular and Molecular Medicine 28, no. 23 (2024): e70222, https://onlinelibrary.wiley.com/doi/10.1111/jcmm.70222. 39632282 10.1111/jcmm.70222 PMC11617115

[21] J. Peres , E. Davis , S. Mowla , et al., “The Highly Homologous T‐Box Transcription Factors, TBX2 and TBX3, Have Distinct Roles in the Oncogenic Process,” Genes & Cancer 1, no. 3 (2010): 272–282, https://gan.sagepub.com/lookup/doi/10.1177/1947601910365160. 21779450 10.1177/1947601910365160 PMC3092191

[22] S. Prince , T. Wiggins , P. A. Hulley , and S. H. Kidson , “Stimulation of Melanogenesis by Tetradecanoylphorbol 13‐Acetate (TPA) in Mouse Melanocytes and Neural Crest Cells,” Pigment Cell & Melanoma Research 16, no. 1 (2003): 26–34, 10.1034/j.1600-0749.2003.00008.x. 12519122

[23] V. Damerell , M. A. Ambele , S. Salisbury , et al., “The c‐Myc/TBX3 Axis Promotes Cellular Transformation of Sarcoma‐Initiating Cells,” Frontiers in Oncology 11 (2022): 801691, https://www.frontiersin.org/articles/10.3389/fonc.2021.801691/full. 35145908 10.3389/fonc.2021.801691 PMC8821881

[24] T. Willmer , J. Peres , S. Mowla , A. Abrahams , and S. Prince , “The T‐Box Factor TBX3 Is Important in S‐Phase and Is Regulated by c‐Myc and Cyclin A‐CDK2,” Cell Cycle 14, no. 19 (2015): 3173–3183, https://www.tandfonline.com/doi/full/10.1080/15384101.2015.1080398. 26266831 10.1080/15384101.2015.1080398 PMC4825571

[25] J. S. Bleloch , A. du Toit , L. Gibhard , et al., “The Palladacycle Complex AJ‐5 Induces Apoptotic Cell Death While Reducing Autophagic Flux in Rhabdomyosarcoma Cells,” Cell Death Discovery 5, no. 1 (2019): 60, https://www.nature.com/articles/s41420‐019‐0139‐9. 30701092 10.1038/s41420-019-0139-9 PMC6349869

[26] N. Fraunhoffer , C. Teyssedou , P. Pessaux , M. Bigonnet , N. Dusetti , and J. Iovanna , “Development of Transcriptomic Tools for Predicting the Response to Individual Drug of the mFOLFIRINOX Regimen in Patients With Metastatic Pancreatic Cancer,” Frontiers in Oncology 14 (2024): 1437200, https://www.frontiersin.org/articles/10.3389/fonc.2024.1437200/full. 39323995 10.3389/fonc.2024.1437200 PMC11422012

[27] H.‐J. Son and A. Moon , “Epithelial‐Mesenchymal Transition and Cell Invasion,” Toxicological Research 26, no. 4 (2010): 245–252, https://link.springer.com/10.5487/TR.2010.26.4.245. 24278531 10.5487/TR.2010.26.4.245 PMC3834497

[28] P. Chen , D. Tian , and M. Liu , “The Role of Tbx2 in Pancreatic Cancers and Its Regulation by Wnt/β‐Catenin Signaling,” Chinese‐German Journal of Clinical Oncology 7, no. 7 (2008): 404–409, 10.1007/s10330-008-0054-7.

[29] S. Duo , T. Tiao‐Dong , Z. Lei , W. Wei , S. Hong‐Li , and D. Xian‐Wei , “Expression and Clinical Significance of tbx2 in Pancreatic Cancer,” Asian Pacific Journal of Cancer Prevention 10, no. 1 (2009): 118–122, http://www.ncbi.nlm.nih.gov/pubmed/19469638. 19469638

[30] J. Li , D. Ballim , M. Rodriguez , et al., “The Anti‐Proliferative Function of the TGF‐β1 Signaling Pathway Involves the Repression of the Oncogenic TBX2 by Its Homologue TBX3,” Journal of Biological Chemistry 289, no. 51 (2014): 35633–35643, https://linkinghub.elsevier.com/retrieve/pii/S002192581956094X. 25371204 10.1074/jbc.M114.596411 PMC4271245

[31] T.‐J. Oh , A. Adhikari , T. Mohamad , A. Althobaiti , and J. Davie , “TBX3 Represses TBX2 Under the Control of the PRC2 Complex in Skeletal Muscle and Rhabdomyosarcoma,” Oncogene 8, no. 4 (2019): 27, http://www.nature.com/articles/s41389‐019‐0137‐z. 10.1038/s41389-019-0137-z PMC6461654 30979887

[32] L. Mohamed , S. Chakraborty , K. N. ArulJothi , et al., “Galenia Africana Plant Extract Exhibits Cytotoxicity in Breast Cancer Cells by Inducing Multiple Programmed Cell Death Pathways,” Saudi Pharmaceutical Journal 28, no. 10 (2020): 1155–1165, https://linkinghub.elsevier.com/retrieve/pii/S1319016420301754. 33132708 10.1016/j.jsps.2020.08.004 PMC7584788

[33] P. P. Kumar , U. Emechebe , R. Smith , et al., “Coordinated Control of Senescence by lncRNA and a Novel T‐Box3 Co‐Repressor Complex,” eLife 3 (2014): e02805, https://elifesciences.org/articles/02805. 24876127 10.7554/eLife.02805 PMC4071561

[34] T. Willmer , S. Hare , J. Peres , and S. Prince , “The T‐Box Transcription Factor TBX3 Drives Proliferation by Direct Repression of the p21WAF1 Cyclin‐Dependent Kinase Inhibitor,” Cell Division 11, no. 1 (2016): 6, 10.1186/s13008-016-0019-0. 27110270 PMC4840944

[35] G. Prelich , “Gene Overexpression: Uses, Mechanisms, and Interpretation,” Genetics 190, no. 3 (2012): 841–854, https://academic.oup.com/genetics/article/190/3/841/6062439. 22419077 10.1534/genetics.111.136911 PMC3296252

[36] P. Wang , Z. Zhou , A. Hu , et al., “Both Decreased and Increased SRPK1 Levels Promote Cancer by Interfering With PHLPP‐Mediated Dephosphorylation of Akt,” Molecular Cell 54, no. 3 (2014): 378–391, https://linkinghub.elsevier.com/retrieve/pii/S1097276514002123. 24703948 10.1016/j.molcel.2014.03.007 PMC4019712

[37] W. Fan , X. Huang , C. Chen , J. Gray , and T. Huang , “TBX3 and Its Isoform TBX3 + 2a Are Functionally Distinctive in Inhibition of Senescence and Are Overexpressed in a Subset of Breast Cancer Cell Lines,” Cancer Research 64, no. 15 (2004): 5132–5139, https://aacrjournals.org/cancerres/article/64/15/5132/511525/TBX3‐and‐Its‐Isoform‐TBX3‐2a‐Are‐Functionally. 15289316 10.1158/0008-5472.CAN-04-0615

[38] M. Joshi , P. Dey , and A. De , “Recent Advancements in Targeted Protein Knockdown Technologies—Emerging Paradigms for Targeted Therapy,” Exploration of Targeted Anti‐Tumor Therapy 4, no. 6 (2023): 1227–1248, https://www.explorationpub.com/Journals/etat/Article/1002194. 38213543 10.37349/etat.2023.00194 PMC10776596

[39] K. Schelch , L. Vogel , A. Schneller , et al., “EGF Induces Migration Independent of EMT or Invasion in A549 Lung Adenocarcinoma Cells,” Frontiers in Cell and Developmental Biology 9 (2021): 634371, https://www.frontiersin.org/articles/10.3389/fcell.2021.634371/full. 33777943 10.3389/fcell.2021.634371 PMC7994520

[40] D. Schaeffer , J. A. Somarelli , G. Hanna , G. M. Palmer , and M. A. Garcia‐Blanco , “Cellular Migration and Invasion Uncoupled: Increased Migration Is Not an Inexorable Consequence of Epithelial‐To‐Mesenchymal Transition,” Molecular and Cellular Biology 34, no. 18 (2014): 3486–3499, https://www.tandfonline.com/doi/full/10.1128/MCB.00694‐14. 25002532 10.1128/MCB.00694-14 PMC4135620

[41] X. Zheng , J. L. Carstens , J. Kim , et al., “Epithelial‐To‐Mesenchymal Transition Is Dispensable for Metastasis but Induces Chemoresistance in Pancreatic Cancer,” Nature 527, no. 7579 (2015): 525–530, https://www.nature.com/articles/nature16064. 26560028 10.1038/nature16064 PMC4849281

[42] E. H. Mahlamäki , M. Bärlund , M. Tanner , et al., “Frequent Amplification of 8q24, 11q, 17q, and 20q‐Specific Genes in Pancreatic Cancer,” Genes, Chromosomes & Cancer 35, no. 4 (2002): 353–358, https://onlinelibrary.wiley.com/doi/10.1002/gcc.10122. 12378529 10.1002/gcc.10122

[43] C. M. Fillmore , P. B. Gupta , J. A. Rudnick , et al., “Estrogen Expands Breast Cancer Stem‐Like Cells Through Paracrine FGF/Tbx3 Signaling,” Proceedings of the National Academy of Sciences of the United States of America 107, no. 50 (2010): 21737–21742, https://pnas.org/doi/full/10.1073/pnas.1007863107. 21098263 10.1073/pnas.1007863107 PMC3003123

[44] R. Tasaka , T. Fukuda , M. Shimomura , et al., “TBX2 Expression Is Associated With Platinum‐Sensitivity of Ovarian Serous Carcinoma,” Oncology Letters 15 (2017): 3085–3090, http://www.spandidos‐publications.com/10.3892/ol.2017.7719. 29435041 10.3892/ol.2017.7719 PMC5778828

[45] Y. Luo , L. Z. Ellis , K. Dallaglio , et al., “Side Population Cells From Human Melanoma Tumors Reveal Diverse Mechanisms for Chemoresistance,” Journal of Investigative Dermatology 132, no. 10 (2012): 2440–2450, https://linkinghub.elsevier.com/retrieve/pii/S0022202X15354804. 22622430 10.1038/jid.2012.161 PMC3434242

[46] F. Yi , J. Du , W. Ni , and W. Liu , “Tbx2 Confers Poor Prognosis in Glioblastoma and Promotes Temozolomide Resistance With Change of Mitochondrial Dynamics,” Oncotargets and Therapy 10 (2017): 1059–1069, https://www.dovepress.com/tbx2‐confers‐poor‐prognosis‐in‐glioblastoma‐and‐promotes‐temozolomide‐‐peer‐reviewed‐article‐OTT. 28260920 10.2147/OTT.S124012 PMC5325101

[47] J. O. Humtsoe , E. Koya , E. Pham , et al., “Transcriptional Profiling Identifies Upregulated Genes Following Induction of Epithelial‐Mesenchymal Transition in Squamous Carcinoma Cells,” Experimental Cell Research 318, no. 4 (2012): 379–390, https://linkinghub.elsevier.com/retrieve/pii/S001448271100468X. 22154512 10.1016/j.yexcr.2011.11.011

[48] L. Zhang , L. E. Pitcher , V. Prahalad , L. J. Niedernhofer , and P. D. Robbins , “Targeting Cellular Senescence With Senotherapeutics: Senolytics and Senomorphics,” FEBS Journal 290, no. 5 (2023): 1362–1383, https://febs.onlinelibrary.wiley.com/doi/10.1111/febs.16350. 35015337 10.1111/febs.16350

[49] J. L. Kirkland and T. Tchkonia , “Senolytic Drugs: From Discovery to Translation,” Journal of Internal Medicine 288, no. 5 (2020): 518–536, https://onlinelibrary.wiley.com/doi/10.1111/joim.13141. 32686219 10.1111/joim.13141 PMC7405395

[50] S. Jaber , M. Warnier , C. Leers , et al., “Targeting Chemoresistant Senescent Pancreatic Cancer Cells Improves Conventional Treatment Efficacy,” Molecular Biomedicine 4, no. 1 (2023): 4, 10.1186/s43556-023-00116-4. 36739330 PMC9899302

