# Supplementary material for: The T‐Box Transcription Factors TBX2 and TBX3 Are Molecular Targets of Piroctone Olamine in the Treatment of Pancreatic Cancer
Source: J Cell Mol Med. 2025 Jul 27;29(14):e70736. doi: 10.1111/jcmm.70736 (PMC12301173; doi:10.1111/jcmm.70736)
Supplement: Supplementary file 1 — Table S1. Western blotting antibody list. Table S2. Immunofluorescence antibody list. [file JCMM-29-e70736-s002.docx]

**Table S1: Western blotting antibody list**

| **Antibody** | **Supplier** | **Dilution** |
| --- | --- | --- |
| TBX3 | Cloud-Clone Corp | 1:1000 |
| Phospho-Histone H2A.X (Ser139) | Cell Signaling Technology | 1:1000 |
| Poly (ADP-ribose) polymerase (PARP) | Cell Signaling Technology | 1:1000 |
| Caspase-9 | Cell Signaling Technology | 1:1000 |
| Microtubule-associated protein light chain 3 (LC3) | Cell Signaling Technology | 1:1000 |
| Vimentin (R28) | Cell Signaling Technology | 1:1000 |
| Cleaved Caspase-7 (Asp198) (D6H1) | Cell Signaling Technology | 1:1000 |
| β-catenin (D10A8) | Cell Signaling Technology | 1:1000 |
| Cyclin B1 (V152) | Cell Signaling Technology | 1:1000 |
| Caspase-8 (1C12) | Cell Signaling Technology | 1:1000 |
| SQSTM1/p62 (D5L7G) | Cell Signaling Technology | 1:1000 |
| E-cadherin (4A2) | Cell Signaling Technology | 1:1000 |
| N-cadherin (13A9) | Cell Signaling Technology | 1:1000 |
| MMP2 | Cell Signaling Technology | 1:500 |
| MMP9 | Cell Signaling Technology | 1:1000 |
| TIMP3 | Cell Signaling Technology | 1:1000 |
| CDK2 | Santa Cruz Biotechnology | 1:1000 |
| Twist | Santa Cruz Biotechnology | 1:1000 |
| Flag | Santa Cruz Biotechnology | 1:1000 |
| Cyclin A (H-432) | Santa Cruz Biotechnology | 1:1000 |
| p21 | Santa Cruz Biotechnology | 1:500 |
| TBX2 | Santa Cruz Biotechnology | 1:500 |
| β-actin | Santa Cruz Biotechnology | 1:3000 |
| Horseradish peroxidase (HRP)-conjugated goat anti-rabbit | Biorad | 1:3000 |
| HRP-goat anti-mouse | Biorad | 1:3000 |

**Table S2: Immunofluorescence antibody list**

| **Antibody** | **Supplier** | **Dilution** |
| --- | --- | --- |
| Phospho-Histone H2A.X (Ser139) | Cell Signaling Technology | 1:100 |
| Microtubule-associated protein light chain 3 (LC3) | Cell Signaling Technology | 1:200 |
| Cy3 | Jackson Immuno Research Laboratories Inc. | 1:500 |
