# Supplementary figures and images for: The T‐Box Transcription Factors TBX2 and TBX3 Are Molecular Targets of Piroctone Olamine in the Treatment of Pancreatic Cancer
Source: J Cell Mol Med. 2025 Jul 27;29(14):e70736. doi: 10.1111/jcmm.70736 (PMC12301173; doi:10.1111/jcmm.70736)

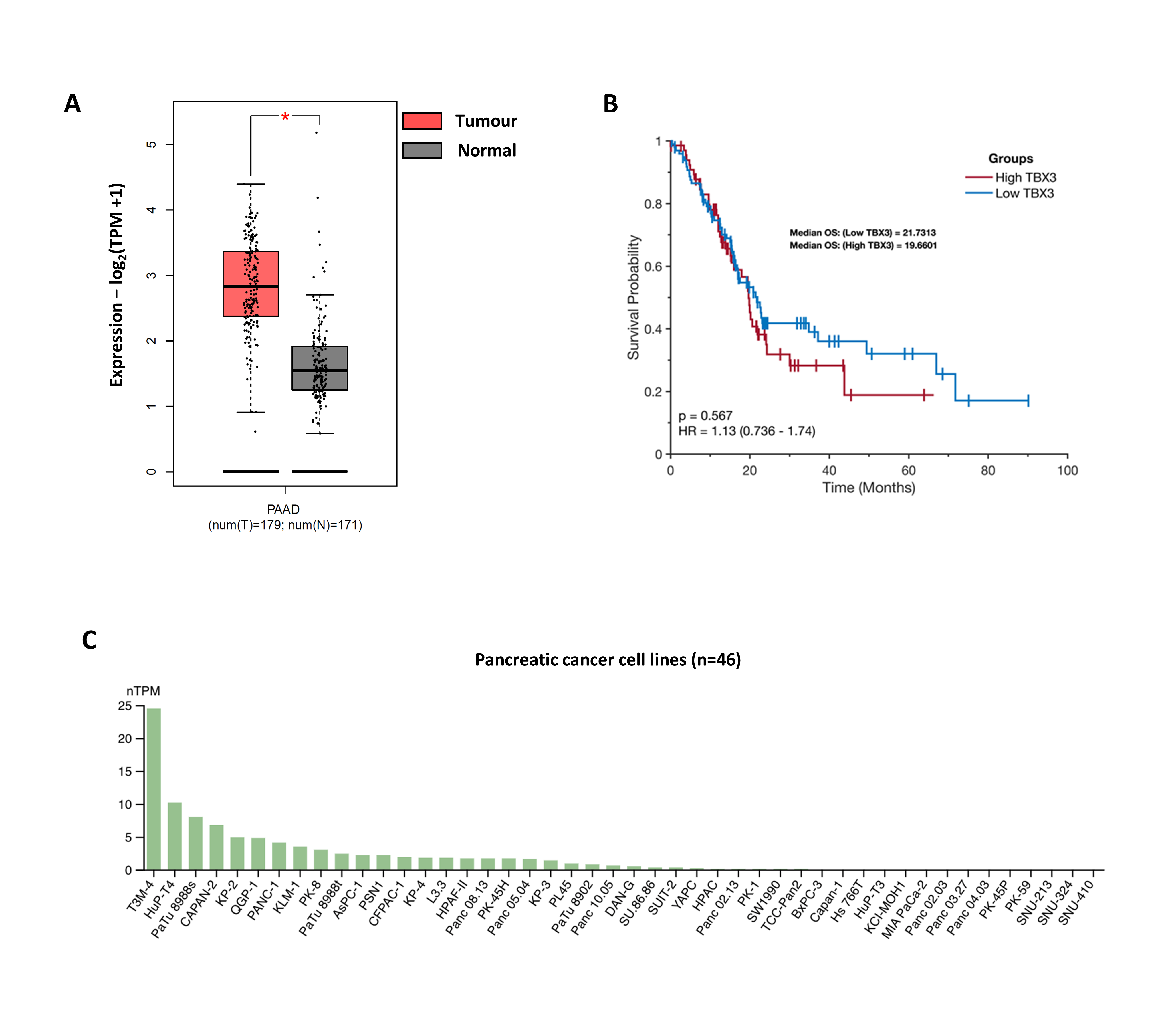

Supplement: Supplementary file 2 — Figure S1. The expression of TBX3 and association with patient survival in PDAC. (A) Analysis of The Cancer Genome Atlas PDAC cohort (TCGA‐PAAD) database for TBX3 levels in PDAC patient tissues and adjacent normal tissues. (B) Kaplan–Meier analysis of the survival rates between PDAC tumours that express high TBX3 and those that express low TBX3. (C) Analysis of the Cancer Cell Line Encyclopaedia (CCLE) database for TBX3 levels in PDAC cell lines (n = 46). [file JCMM-29-e70736-s001.tif]
